# Improving Emergency Department Door to Doctor Time and Process Reliability: A Successful Implementation of Lean Methodology

**DOI:** 10.1097/MD.0000000000001679

**Published:** 2015-10-23

**Authors:** Mazen J. El Sayed, Ghada R. El-Eid, Miriam Saliba, Rima Jabbour, Eveline A. Hitti

**Affiliations:** From the Department of Emergency Medicine, American University of Beirut Medical Center, Beirut, Lebanon.

## Abstract

The aim of this study is to determine the effectiveness of using lean management methods on improving emergency department door to doctor times at a tertiary care hospital.

We performed a before and after study at an academic urban emergency department with 49,000 annual visits after implementing a series of lean driven interventions over a 20 month period. The primary outcome was mean door to doctor time and the secondary outcome was length of stay of both admitted and discharged patients. A convenience sample from the preintervention phase (February 2012) was compared to another from the postintervention phase (mid-October to mid-November 2013). Individual control charts were used to assess process stability.

Postintervention there was a statistically significant decrease in the mean door to doctor time measure (40.0 minutes ± 53.44 vs 25.3 minutes ± 15.93 *P* < 0.001). The postintervention process was more statistically in control with a drop in the upper control limits from 148.8 to 72.9 minutes. Length of stay of both admitted and discharged patients dropped from 2.6 to 2.0 hours and 9.0 to 5.5 hours, respectively. All other variables including emergency department visit daily volumes, hospital occupancy, and left without being seen rates were comparable.

Using lean change management techniques can be effective in reducing door to doctor time in the Emergency Department and improving process reliability.

## INTRODUCTION

Lean management is increasingly being applied in healthcare settings. Applications of this Toyota Production System manufacturing methodology range from small interventions such as patient flow modification or streamlining processes to major facility redesign.^1,2^ More specifically in the Emergency Department (ED), tools from lean-thinking target work structure and processes: the aim is to remove unnecessary steps or waste in the patient's journey through the ED phase of care by reducing waiting times, improving flow, rendering ancillary services more efficient with shorter turnaround times for radiology and laboratory studies, and impacting overall ED length of stay for both admitted and discharged patients.^3^

Key elements for successful implementation of lean tools in the ED include readiness for change, leadership involvement and buy in, engagement of frontline staff or workforce, focus on flow and quality, clear process mapping, and introducing small enhancements that are sustainable over the long term.^4–7^ Such initiatives must be specific to the work setting and take into account patient satisfaction drivers without negatively impacting the employees with work overload, increased stress, or anxiety.^3^

Patient flow through the ED is influenced by both structural and process factors, thereby lending itself well to lean management change application. ED throughput and crowding have been linked to both clinical patient outcomes (time to antibiotics for septic patients or door to balloon time for ST-segment elevation myocardial infarct) as well as service quality.^8–10^ The time from when a patient arrives at the ED, to when they are seen by a provider—door to doctor time—is a component of ED throughput that has been reported to have significant implications on left without being seen rates, with patients overwhelmingly citing prolonged wait times as the reason for leaving prior to assessment.^11^ From a service quality standpoint, door to doctor times is a significant predictor of patient satisfaction.^12,13^

### Goal of Investigation

The goal of this study is to determine the effectiveness of using lean management methodology on improving door to doctor times at a tertiary care hospital.

## METHODS

### Study Design and Setting

This study was conducted at the Emergency Department of the American University of Beirut, the largest tertiary care center in Lebanon, with around 49,000 patient visits per year. Patients are triaged to 3 separate sections of the ED based on their acuities and age: ED1 is the adult high acuity section, ED2 is the adult low acuity section, and ED3 is where all pediatric patients are seen regardless of acuity. The majority of patients present to our ED during the evening shift (4 pm–12 am, 46%), followed by the day shift (8 am–4 pm, 40%), and overnight shift (12 am–8 am, 14%). Registration and payment are done at the beginning of the visit with around 75% of ED visits covered through private insurance coverage, 23% out-of-pocket, and 2% covered through governmental type of coverage. The ED uses a homegrown dashboard system that electronically captures registration time, diagnostic order times, and discharge time from the ED. The dashboard does not capture patient arrival time to the ED nor the time a provider starts seeing the patient.

A pre- and postintervention analysis was carried out comparing 2 cohorts of patients (pre and post) considered as convenience samples of the ED visits. The study was deemed exempt from human subject research by the institutional review board of American University of Beirut and conforms to the Declaration of Helsinki provisions.

### Lean Tools and Interventions

In January 2012, hospital administration identified the ED as a priority performance improvement area and appointed a hospital expert in change management to the ED performance improvement committee which was comprised of the ED chairperson, the ED medical director, 2 nurses including the ED nurse manager, case management, clerks, and registration staff. This Kaizen team, after analyzing the trends in patient comments and complaints, initiated a series of measures using the lean methodology to reduce delays in waiting times and more specifically door to doctor time.

**FIGURE 1:**
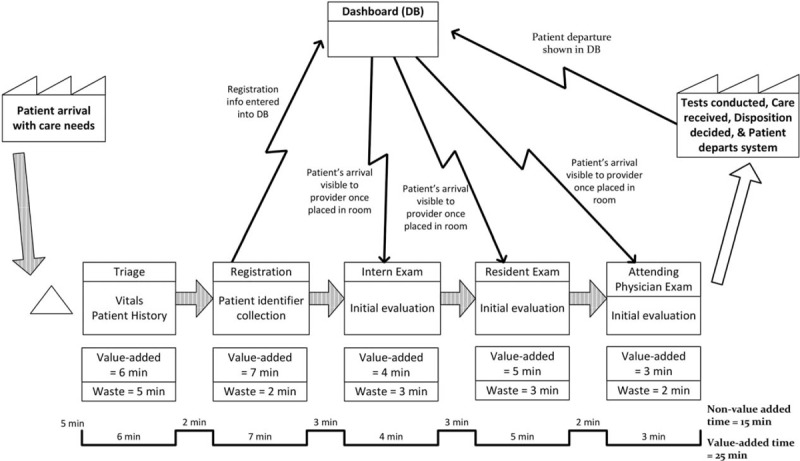
Value stream map of door to doctor phase.

**FIGURE 2:**
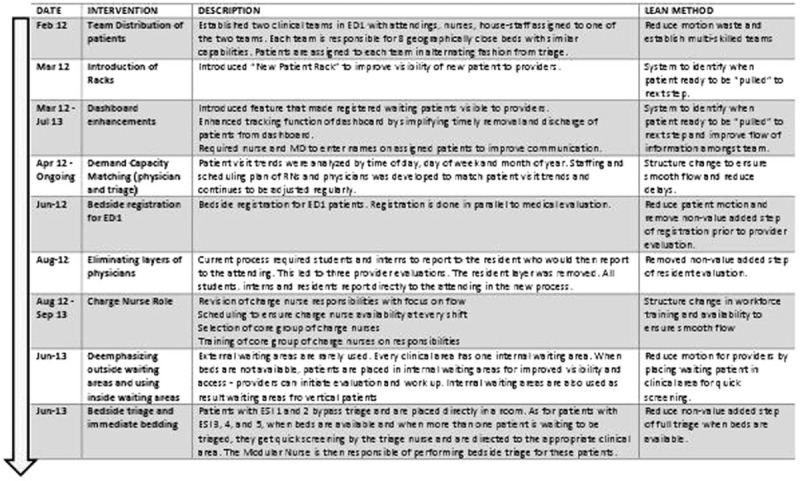
Timeline of value-driven lean interventions to improve door to doctor time.

The team focused on value-driven, rather than expense reducing, lean techniques. The first tool used was process mapping of the patient journey from arrival to being assessed by an attending physician, followed by Value Stream Mapping to identify value-added and nonvalue-added (waste) time in each step (Figure 1). This process was done in an iterative fashion with modifications taking into account the feedback of all those who were involved, particularly front-line staff. Metric baseline was established for door to doctor time and for patient length of stay. Root cause analysis of bottlenecks was completed with the aim of identifying obstacles to continuous flow and eliminating waste from the process. Structural setup was looked at with a focus on optimizing worker factors (responsibilities of staff), organizational factors (staffing), communication systems (dashboard-tracking system), and physical environment. Figure 2 includes a summary of the key interventions that were identified and implemented over 20 months through short-cycle continuous improvement sessions by the Kaizen team that met bimonthly to implement, assess, and modify the processes.

### Methods, Measurement, and Outcomes

Prior to initiating the interventions, a convenience sample of 252 patients in February 2012 was tracked by 2 research assistants who documented the following times: patient arrival time (the time patient entered the ED door), student time, intern time, resident time, and attending time (provider times was taken as soon as the provider began communicating with the patient). Similarly, after completing the interventions, another convenience sample of 135 patients was tracked by the same 2 research assistants from mid-October to mid-November 2013. In both phases, the sample covered patients from all 3 shifts of the day and all days of the week. The primary outcome was door to doctor time, which was calculated from the time the patient walked through the ED door until the time an attending physician saw the patient.

Length of stay of patients in the ED was a secondary outcome and was retrieved from the electronic business intelligence software QlikView that calculates length of stay as the difference between electronically captured registration time and the electronically captured time stamp of discharge from the ED. Other variables including daily number of ED visits, gender, left without being seen rate, and hospital occupancy were also extracted from Qlikview for the total ED population during the study period. Number of ED-related patient complaints for the period 6 months prior to the study and immediately 6 months poststudy were retrieved from the hospital Patient Affairs office where all patient concerns are referred to and processed. Patients who left without completing registration were excluded from the study population.

### Analysis

The Statistical Package for Social Sciences, version 21.0, was used for data entry and analyses. To compare the 2 intervention phases, Student's *t*-test and Pearson Chi-square test were used for continuous and categorical variables, respectively. Additional nonparametric testing (Mann–Whitney *U* test) was done to compare door to doctor time in the 2 intervention phases. A *P*-value of <0.05 was used to indicate statistical significance. Individual control chart (I-chart) was used to analyze trends, special cause variations (nonroutine events), common cause variations (routine events), and assess the process for stability (statistical control).

## RESULTS

**FIGURE 3:**
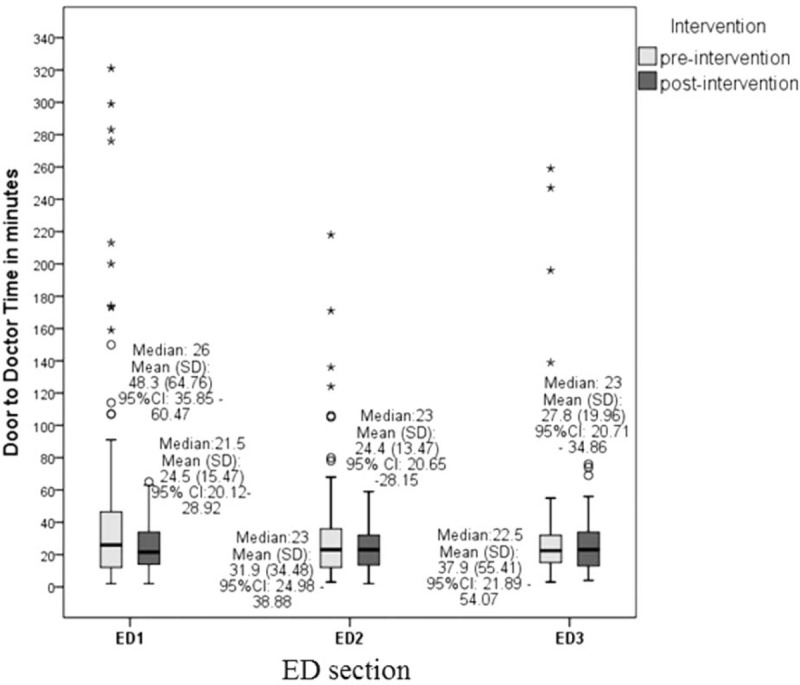
Mean door to doctor time by Emergency Department (ED) section.

**FIGURE 4:**
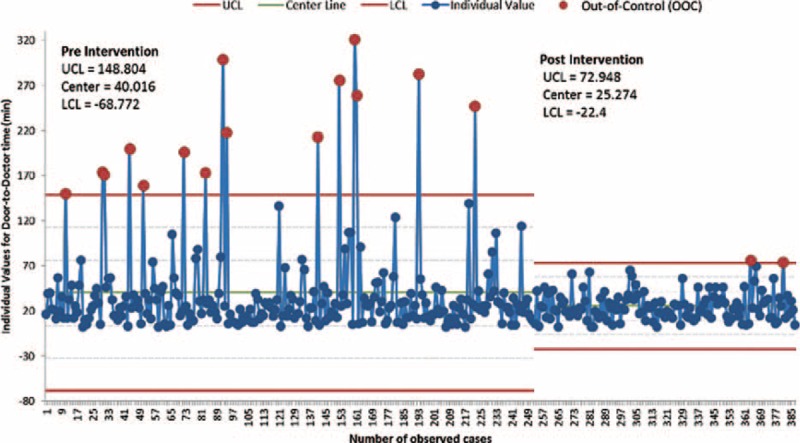
Individual control chart of door to doctor time for pre and post-lean intervention periods.

In the preintervention phase, approximately 8% (252/3126) of the population was sampled compared to approximately 4% (135/3399) in the postintervention phase with similar distribution of patients among ED sections in both samples (Table 1). The lean driven interventions led to significant improvements in mean door to doctor times which dropped by 37% postintervention when compared to preintervention (25.3 vs 40.0 minutes; *P* < 0.001, 95% CI 7.59–21.89) (Table 2). Stratifying by ED section the patient was triaged to, the mean door to doctor time for ED1 was significantly lower in the postintervention as compared to the preintervention (Figure 3). Control chart analysis of the process with time demonstrated a much more controlled process postintervention (Figure 4). In the preintervention phase, there were 15 out of control data points where the door to doctor time exceeded 3 standard deviations from the centerline, whereas, the postintervention phase had only 2 out of control points. The upper control limit dropped to 72.9 minutes postintervention compared to 148.8 minutes preintervention, demonstrating a much more controlled process with less variation. Other ED operation metrics also improved in the postintervention phase including reduction in mean ED length of stay of both admitted and discharged patients. Analysis of total ED population in the pre- and postintervention phase showed no difference in gender or daily ED visit volume. Patients in the postintervention period were however slightly younger (Table 3).

**TABLE 1:**
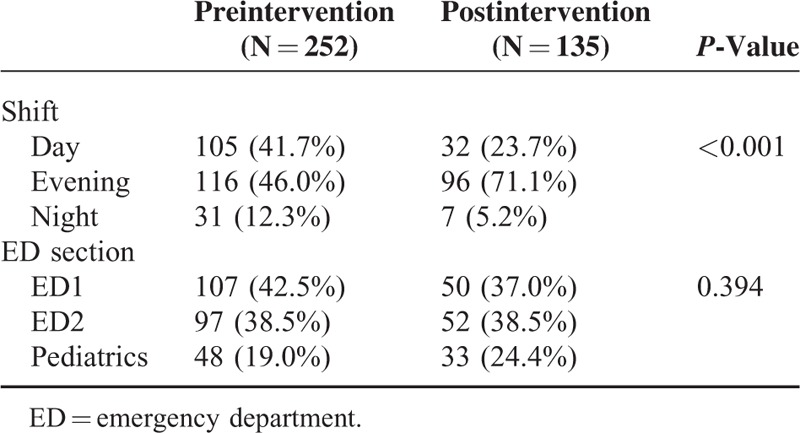
Comparison of Study Sample Pre- and Post-Lean Implementation

**TABLE 2:**
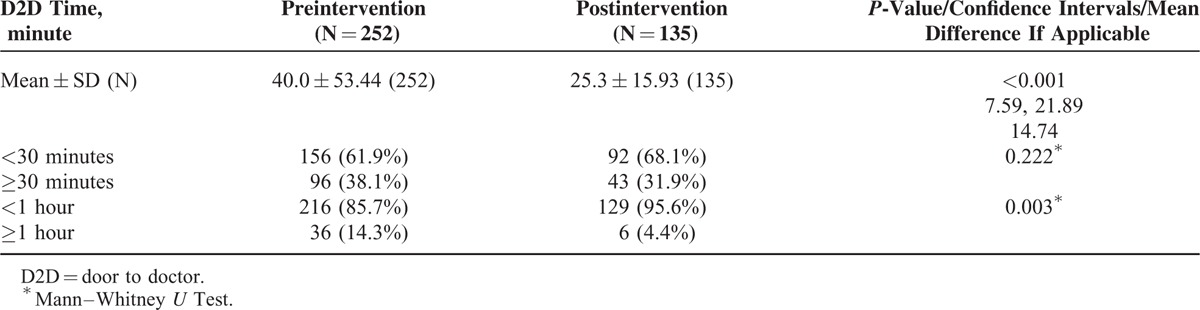
Comparison of Door to Doctor Time Pre- and Postintervention

**TABLE 3:**
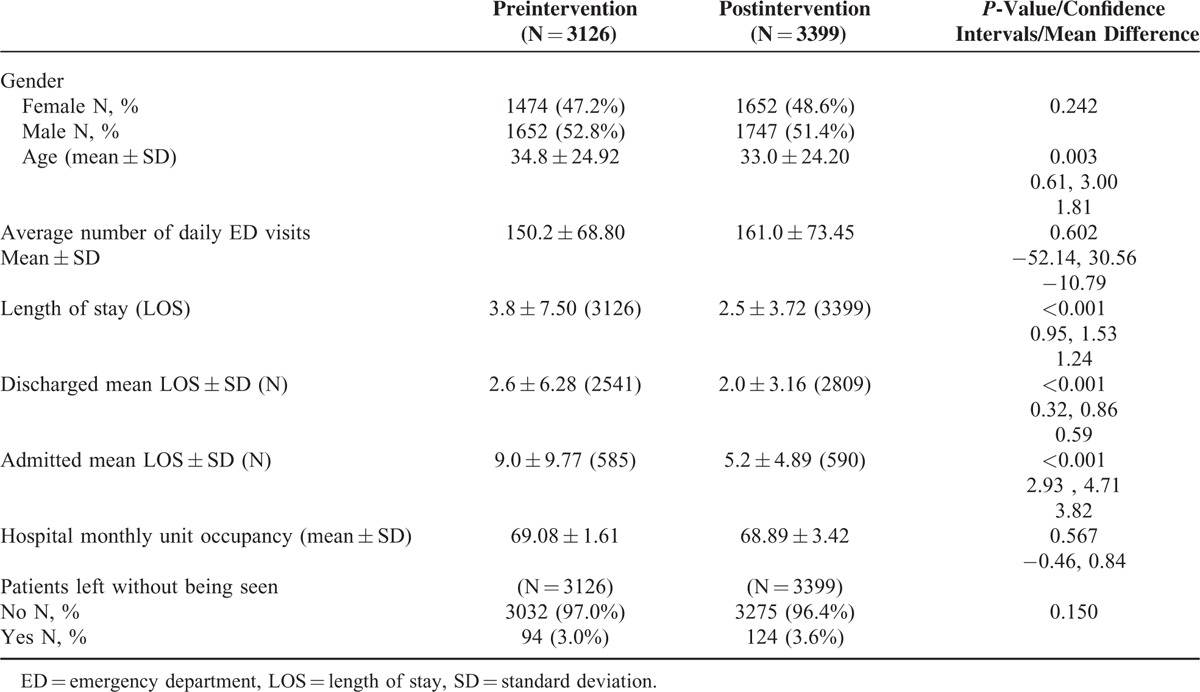
Comparison of Total ED Population During Pre- and Postintervention Period

The change in left without being seen rate was not statistically significant between the 2 study periods. There were no significant changes in the other variables that may impact operations including mean daily ED visit number and hospital occupancy. Although patient complaints per 1000 visits dropped in the 6-month period postintervention from 0.6 to 0.4 (*P* = 0.386, 95% CI 0.27–2.59) compared to the 6-month period prior to the intervention, this was not statistically significant.

## DISCUSSION

In our setting, lean methodology was effective in reducing door to doctor time through the implementation of a series of small changes that were compatible with the existing ED physical structure without facility redesign or major expenses. Postintervention, we noticed an overall improvement of our ED operations including a reduction in the ED mean length of stay for both admitted and discharged patients.

Other studies reported different lean tool implementation in the ED such as streaming of patients based on their predicted outcomes (admission vs discharge), but were less effective in reducing door to doctor times.^7^ Studies that looked at creating fast tracks for specific low acuity patients showed reduced waiting times, however only for low complexity patients.^13,14^ In our study, door to doctor time was reduced in all sections but was statistically significant for ED1 where delays were greatest preintervention. Furthermore, the 31.6% drop of door to doctor time in our study was bigger than previously reported, especially that we looked at the time to being seen by an attending physician and not by a house staff or other medical personnel.^7,13–15^

Our study is the first to look at impact of using lean methodology on reliability of the ED intake process as measured by door to doctor time. The use of control charts in healthcare to evaluate the impact of quality improvement projects on reliability of processes is growing, allowing managers to detect changes using fewer data while still maintaining statistical rigor.^16^ The narrower upper and lower control limits in the postintervention phase in our study reflect a more statistically controlled process and thus a more reliable and consistent patient experience with respect to door to doctor times across all sections. The drop in out of control points in the postintervention phase also reflects fewer special cause variations that were reducing process predictability in the preintervention phase. The end-result of the cumulative changes thus reflect a higher-reliability process that is increasingly recognized as an essential aspect of safer and higher quality care.^17^

Although our key intervention was using lean methodology to develop and implement multiple customized changes, there are several initiatives worth highlighting for generalizability potential. One of the main components of our quality improvement plan was demand capacity matching: a dynamic-scheduling plan was developed by closely analyzing historical trends in hourly, daily, and monthly volumes and then matching the scheduling of medical and nursing staff accordingly to ensure appropriate staffing. In addition, we attempted to address bed capacity constraints by introducing internal waiting areas in each of the sections that, in peak times, serve as result waiting areas as well as additional space for initial medical evaluation pending availability of treatment rooms. These 2 interventions specifically may be applicable to other ED settings.

The success of our lean program can be attributed to several factors that are essential for successful lean initiatives in the ED.^3^ Our department had an already active multidisciplinary process improvement committee that was ready for change. We ensured engagement from all stakeholders by expanding our team to include representatives from all front-liners. In addition, discussion of interventions was systematically integrated into both the nursing and medical staff monthly meetings, thus allowing for direct timely feedback throughout all phases of the change process. We also secured top management support for our initiatives including the help of an expert in change management. We adapted lean to our local context and evaluated every intervention regularly making small modifications throughout to ensure optimization.

Although our left without being seen rates did not change, this measure reflects processes and factors that are specific to our setting where financial clearance is done at registration for low complexity cases. Charity care and other alternatives are available for high acuity cases regardless of ability to afford care and are not part of the left without being seen cases. Nevertheless, insurance coverage denial for the ED low acuity visits accounts for most of the cases that leave without completing registration as compared to the United States experience where waiting times comprise the greatest barrier to assessment by a physician in the ED.^18–20^

## LIMITATIONS

The use of convenience samples to examine the impact of our interventions is one key limitation of our study. Although we ensured shift selection that matched the distribution of patient visits in the preintervention phase, we were unable to do this in the postintervention phase because of limitations in our research assistant availability for the overnight shift. Furthermore, our sample size was small and this may have limited our ability to detect statistically significant differences in pre- and postintervention times. In addition, we had to rely on manual collection of both “door time” and “doctor time” since these time stamps are not captured electronically by our information system. Finally, although we were able to retrieve and detect a slight improvement in ED-related patient complaints, we did not directly assess the impact of the decrease in door to doctor time on patient satisfaction.

## CONCLUSIONS

In summary, lean methodology was implemented successfully in our setting and improved on door to doctor time and process reliability. The Kaizen team focused on a series of small interventions that were based on heavy frontline involvement and continuous enhancements. Other ED managers can easily adopt the strategies that we described with minor modifications that take into account the existing culture and environment specific to their setting. Value-focus, administrative support, and heavy frontline involvement are all essential for the successful implementation of lean methods in the ED.
